# Supplementary material for: SyNDI: synchronous network data integration framework
Source: BMC Bioinformatics. 2018 Nov 6;19:403. doi: 10.1186/s12859-018-2426-5 (PMC6219086; doi:10.1186/s12859-018-2426-5)
Supplement: Supplementary file 5 — Files of the example presented in Additional file 4. This additional file is a zip package that contains all files used in Additional file 4. Detailed descriptions of each of these files are presented in a README file included in this zip package. (GZ 30411 kb) [file 12859_2018_2426_MOESM5_ESM.gz › SyNDITools/Meme2Fimo/meme_4.9.0_4.tar.gz!/meme_4.9.0/doc/psp-gen.xml]

psp-gen


# psp-gen

**Usage:** `psp-gen -pos <filename> -neg <filename> [options]`

**Short description:**

`psp-gen` is used to allow MEME to perform discriminative
motif discovery—to find motifs overrepresented in one set of
sequences compared to in another set. It takes two files as
input—the sequence file to be input to MEME, (the "positive"
file) and a "negative" sequence file of sequences believed not to
contain the same motifs as in the "positive"
file. `psp-gen` creates a file for use by MEME that
encapsulates information about probable discriminative motifs.
`psp-gen` records its chosen motif width in the file,
and MEME is able to adjust the data when it tries different motif
widths.

**Input:**

- `-pos <positive filename>` – specifies a file containing
  sequences in FASTA format, to be
  used in the PSP calculation as the positive set.
- `-neg <negative filename>` – specifies a file containing
  sequences in FASTA format, to be
  used as the negative set in PSP calculation.

You should choose the negative set as a contrast to the positive set:
similar in most respects, but differing in the features that you would
like to associate with motifs.

**Output:**

- Writes to standard output.
- Writes to standard error if `-reportscores`
  or `-verbose` options are set.

The output contains an entry for every sequence in the original data,
in a FASTA-like format. The original FASTA name lines are retained,
with additional annotation indicating the motif width and the scaling
applied to raw scores.

Only the name and the motif width are interpreted by MEME; the scaling
information is recored as documentation of the run of `psp-gen`
that generate the PSP file.

Instead of sequence data, the output contains a
number for each position in the sequence, representing the probability
that a motif starts at that position. For example:

```
>A2ACQ1 3 scaledmin = 0.1 scaledmax = 0.9
0.001514752 0.001571433 0.00130429 0.002221764 0.00194399
```

starts with a name line indicating that the original sequence is called
"`A2ACQ1`", `psp-gen` found the "best" motif
scores with a width of 3, and scores were scaled to 0.1..0.9, before
converting them to probabilities. The next
line contains the first 5 positional prior values for
sequence `A2ACQ1`.

**Options:**

Options are all case-specific:

- `-h` – print a usage message and quit.
- `-minw <W>` – minimum width to use when selecting the
  "best" width for PSPs.  
  Default: 4.
- `-maxw <W>` – maximum width to use when selecting the
  "best" width for PSPs.  
  Default: 20.
- `-alpha DNA|prot` – choose the sequence alphabet.  
  Default: DNA.
- `-triples` – Use spaced triples instead of whole-word
  matches. Recommended for protein.  
  Default: use whole words.
- `-fixedstart` – for triples, only consider triples starting
  at the start of the site.  
  Default: allow triples to start anywhere in a width *w* site.
- `-equiv "<letter options>"` – Any sequence of letters
  that appears together should be matched as equal. Separate letter
  groups using "-", e.g. `-equiv "IVL-HKR"` means treat all
  occurrences of `I`, `V` or `L` as the
  same, and all occurrences of `H`, `K`
  or `R` as the same.  
  Default: Treat every letter as unique.
- `-revcomp` – DNA only: score both strands.  
  Default: only score on one strand.
- `-scalemin <N>` – lowest score value after
  scaling.  
  Default: 0.1, unless `scalemax` is set, in which
  case the default is `1-scalemax`
- `-scalemax <N>` – highest score value after
  scaling.  
  Default: 0.9, unless `scalemin` is set, in which
  case the default is `1-scalemin`
- `-maxrange` – choose `w` with the biggest
  *difference* between minimum and maximum scores (before
  scaling).  
  Default: choose `w` with the biggest maximum score (before
  scaling).
- `-raw` – Output scores instead of PSPs.
- `-reportscores` – send to standard error positive and negative file
  names, min and max scores and min and max `w`,
  tab-separated.  
  Default: do not report to standard error.
- `-verbose` – send detailed stats to standard error, reporting
  frequency of each score  
  Default: do not report.

**Examples:**

In each example, the sequence file is the same as the postive set used
for `psp-gen`. While there is no check that you have done
this because MEME runs as a separate program (other than that the
sequence names need to match, as do the number of sites), using a
positive set different to the sequence set MEME searches is unlikely
to be useful.

Generate a discriminative prior for a DNA sequence set
(`pos-dna.fasta`) likely to contain TF binding sites, given
another DNA sequence set unlikely to contain TF binding sites
(`neg-dna.fasta`):   
`psp-gen -pos pos-dna.fasta -neg neg-dna.fasta > dna.psp`

Use these position-specific priors in MEME, searching both strands:  
`meme pos-dna.fasta -psp dna.psp -oc meme_out_dna
-revcomp`

Generate a discriminative prior for a protein sequence set
(`pos-prot.fasta`) associated with a specific function, given
another protein sequence set unlikely to relate to that function
(`neg-prot.fasta`):   
`psp-gen -pos pos-prot.fasta -neg neg-prot.fasta -alpha prot
-triples -maxrange > prot.psp`

Use these position-specific priors in MEME:  
`meme pos-prot.fasta -psp prot.psp -oc meme_out_prot`

View usage information:  
`psp-gen -h`

**Detailed description:**

The `psp-gen` program generates a position-specific
prior (PSP) in a data file, which can be used as an additional input
to MEME to bias the search to sites more likely to
contain a motif (or motifs) of interest. Without using a PSP, MEME
assigns the same prior probability to all sites. A PSP is generated
by `psp-gen` using two sequence sets, each
in FASTA format:

- the *positive set* (or *X*) contains the same
  sequences as you intend to search for a motif using MEME
- the *negative set* (or *Y*) is a contrasting set of
  sequences that is unlikely to contain the motif or motifs of
  interest, but is otherwise similar to the positive set.

The basic principle of a discriminative prior is to start from the
question:

> What fraction of *all* instances of a *w*-wide
> subsequence (or *w*-mer) across both sequence sets occur *in the
> positive set*?

The intuition is that any *w*-mer that is common
in the positive set but not the negative set could form part of motif
of interest, based on the way the two sets are chosen.

PSP calculation starts from the following equation based on the
*D*-prior described in
the Narlikar *et al*. *RECOMB* 2007 paper
"Nucleosome
Occupancy Information Improves *de novo* Motif
Discovery". We count the number of
instances *X*_*i*,*j*_(*w*) of the
subsequence *w* wide starting at position *j* in sequence
*X_i_* of the positive set, and similarly
count *Y*_*i*,*j*_(*w*) in the negative
set, then calculate for each site:

|  |  |  |  |
| --- | --- | --- | --- |
| *D*_*i*,*j*_ | = |  | *X*_*i*,*j*_(*w*) |
|  |  |  | *X*_*i*,*j*_(*w*) + *Y*_*i*,*j*_(*w*) |

As pseudocounts, we add 1 to the enumerator, and 1 +
|*X*|/|*Y*| to the denominator. The purpose of the
pseudocounts is to avoid giving a high score to infrequent
subsequences that are not (or rarely) found in the negative set. Once
we have scored all sites for a given value of `w`, we scale
scores to a range that can be set on the command
line, with the default range `[0.1,0.9]`.

We repeat scoring for the whole range of motif widths under
consideration, and choose a "best" width using one of two methods:
either we choose the value of `w` that maximizes the score
over all sequences, or the value of `w` that maximizes the
difference between the maximum and minimum score over all
sequences. We also allow an option of using *spaced triples*
instead of whole words to match
subsequences. See Variations below for more
details on triples and choosing the "best" width.

Once we have scored every available site and chosen a specific value
of `w`, we convert the scores to
probabilities, based on the following proportionalities,
where *Z_i_* = *j* means that the site for a motif
in sequence *X_i_* is found at location *j* (with *j* = 0
signifying there is no site in the given sequence)
and *D*_*i*,*j*_ is the score for
sequence *X_i_* at the site starting at
location *j*, numbered from 1:

|  |  |  |
| --- | --- | --- |
| *P*(*Z_i_* = 0) | ∝ | 1 |
| and | | |
| *P*(*Z_i_* =*j*) | ∝ | *D*_*i*,*j*_ |
|  |  | 1-*D*_*i*,*j*_ |

We
convert these proportionalities to a PSP by normalizing them to add up
to 1 over each sequence.

***Running with MEME:***

Since MEME runs as a separate program, there is no way to check that
the positive set is the same as the sequence set given to MEME. There
are however checks that every name in the PSP file matches a name in
the MEME sequence set, and that the number of sites in the PSP file
for a particular sequence name matches the number of sites in the
sequence file seen by MEME. While it does not make sense to use a
completely different sequence set for PSP generation and for running
MEME (with the resulting PSP file), MEME can use a PSP file that does
not provide probabilities for every sequence. Any sequences not in the
PSP file are given a uniform prior
probability. MEME documentation
includes more detail on how PSPs are used.

***Variations:***

For each variation, "default" means that you do not need to specify a
command-line switch to turn on that setting.

- *sequence alphabet*: choose `-alpha DNA`
  (default) or `-alpha prot` (protein). For DNA, you also
  have the option to score matches on both strands:
  - `-revcomp` (the default is to score only the given
    strand). This setting applies to both the positive and negative
    sequences.
- *choosing the "best" width*: specifying `-maxw`
  and `-minw` provides limits to `psp-gen` but
  the algorithm chooses one width to generate priors after scoring the
  range of allowed widths, using one of two variations:

- *maximum score* (default): choose the value of *w* that
  maximizes the score over all sequences.
- *maximum range of values* (`-maxrange`):
  choose the value of *w* that maximizes the difference between
  the highest and lowest score over all sequences. This variant is
  useful in data sets where a high number of sites achieve similar
  high scores (more likely to happen with protein sequences).

- *type of word match*:
  - *whole-word match* (default)
  - *spaced triples* (`-triples`): for a case
    where exact-word matches are unlikely to succeed, spaced triples
    are an alternative. Each *w*-wide word is broken down into
    triples containing an initial, middle and final letter, and all
    other letters are treated as don't cares (i.e., they match
    anything). In spaced triples mode, `psp-gen` scores a
    site on the basis of the spaced triple fitting the *w*-wide
    word at that site that has the highest score. For example, the
    subsequence  
    `MTFEKI` contains the following
    triples:  
    `MT...I`  
    `M.F..I`  
    `M..E.I`  
    `M...KI`  
    where "." matches anything when counting matches.
    - An option that only applies to triples
      is `-fixedstart`, which only allows triples for the
      current site under consideration that start at the beginning of the
      site. This option reduces execution time at the expense of
      considering fewer possibilities for scoring a site.
    - default: consider triples of all widths that fit the current site.
- *scaling variations* (`-scalemin` and
  `-scalemax`): the scoring formula creates values in the
  range `[0,1]`, but the conversion to PSPs from scores
  requires values strictly less than 1. Choosing an option other than
  scaling between the default `[0.1,0.9]` reduces (if you
  narrow the range) or increases (if you widen the range) sensitivity
  to small differences in scores. If you only set one
  of `scalemin` or `scalemax`, the other is
  calculated as described in Options.
- *ambiguous characters*: you have two levels of control of
  ambiguous characters:
  - *ambiguous characters in the sequence data*:
    The effect of encountering any of these letters is to score a word
    or triple containing them as if it only occurs once, to minimize its
    score.
  - *treat specific letters as equivalent*: use
    the `-equiv` option to treat specific groups of letters
    as if they were all the same (default: all letters are treated as
    unique). For more detail, see Options.
- *reporting and debugging*: you have various options for
  understanding better how PSPs are calculated.
  - *see raw scores*: `-raw` stops the last stage
    of computation that converts the scores to probabilities; the
    scores sent to standard out are however scaled, using either the
    defaults or selected values of `scalemin`
    and `scalemax` (default: convert scores to PSPs).
  - *report range of scores*:
    see `-reportscores` in Options
    (default: do not report).
  - *see detailed statistics*: `-verbose` causes
    detailed statistics on scores to be sent to standard error
    (default: no statistics reported).

***Performance scalability:***

When you use exact-word (*w*-mer) search, `psp-gen`
run time scales linearly in each of |*X*|, |*Y*| and
`maxw`-`minw`. Formally, time is O((|*X*| +
|*Y*|)(*w_max_* -
*w_min_*)).
For triples, `psp-gen` scales linearly in each of
|*X*| and |*Y*|, and quadratically
in *w_max_*, so run time is O((|*X*| +
|*Y*|)*w_max_*^2^).

Extra memory in both cases scales linearly with |*X*| +
|*Y*|.

Since triples have a higher-order term in time complexity, we predict
run time scalability in more detail than for exact word
match. Adding in constants and a linear term, time complexity
for triples is approximated by (where *W*
= *w_max_*-2, the number of variations in width
used by the algorithm):

|  |  |  |
| --- | --- | --- |
| *t* | = | (*k*_1_\|*X*\| + *k*_2_\|*Y*\|)*W*^2^ + *k*_3_(\|*X*\| + \|*Y*\|)*W* |

On a real machine (iMac, 3.06GHz Intel Core 2 Duo, 6MB L2 cache, 4GB
RAM) the following is a good predictor of the upper bound on run time
for likely values of |*X*|, |*Y*| and range of
widths. Scale any results according to the speed of the actual machine
on which you intend to run `psp-gen`. The formula reflects
runs on randomly generated data; real data will likely result in
faster runs because random data defeats some optimizations
in `psp-gen`.

|  |  |  |
| --- | --- | --- |
| ***t*** | **=** | **(4.37x10^-6^\|*X*\|+ 2.46x10^-06^\|*Y*\|)*W*^2^ + 6.25x10^-06^(\|*X*\| + \|*Y*\|)*W* s** |

For example, for |*X*| = |*Y*| = 60,000
and *w_max_* = 50 (i.e., *W* = 48), estimated run
time = 980s, the same in this case as measured run time. The following
command line reproduces this run:

```
time psp-gen -neg pos60k.fasta -pos neg60k.fasta -alpha prot -minw 3 -maxw 50 -triples > priors60kmaxw50.psp
```

**Known problems:**

- Any letters in sequence represening ambiguous bases or amino acids
  result in a word (*w*-mer) containing that word (or triple in
  the case of protein) being counted whether in the positive or
  negative sets as if it occurred once, to minimize its score.

---

Author: Philip Machanick (p.machanick@imb.uq.edu.au)
